# Impact of imbalanced features on large datasets

**DOI:** 10.3389/fdata.2025.1455442

**Published:** 2025-03-13

**Authors:** Waleed Albattah, Rehan Ullah Khan

**Affiliations:** Department of Information Technology, College of Computer, Qassim University, Buraydah, Saudi Arabia

**Keywords:** machine learning, deep learning, classification, feature extraction, computer vision

## Abstract

The exponential growth of image and video data motivates the need for practical real-time content-based searching algorithms. Features play a vital role in identifying objects within images. However, feature-based classification faces a challenge due to uneven class instance distribution. Ideally, each class should have an equal number of instances and features to ensure optimal classifier performance. However, real-world scenarios often exhibit class imbalances. Thus, this article explores the classification framework based on image features, analyzing balanced and imbalanced distributions. Through extensive experimentation, we examine the impact of class imbalance on image classification performance, primarily on large datasets. The comprehensive evaluation shows that all models perform better with balancing compared to using an imbalanced dataset, underscoring the importance of dataset balancing for model accuracy. Distributed Gaussian (D-GA) and Distributed Poisson (D-PO) are found to be the most effective techniques, especially in improving Random Forest (RF) and SVM models. The deep learning experiments also show an improvement as such.

## 1 Introduction

The imbalance refers to an uneven class instance distribution in image datasets. This can significantly affect the accuracy of Machine Learning models. When the dataset is highly unbalanced, with a large number of samples in the majority class and a small number in the minority class, the models tend to have high accuracy against the majority class but struggle to classify the minority class accurately. This is because there are insufficient examples of minority classes from which the models can learn, and the models are biased toward the majority class.

Various approaches have been developed to tackle this issue, including oversampling methods such as random oversampling, SMOTE, borderline SMOTE, ADASYN, and Deep SMOTE (Barulina et al., 2023). An alternative method involves utilizing a subset of the dataset with missing images in certain classes and adding augmented images. This strategy has demonstrated enhanced metrics when compared to the original imbalanced dataset (Achmad and Haris, 2023).

Addressing the imbalance in image datasets is essential for enhancing the accuracy of machine learning algorithms. The accuracy and reliability of Deep Learning (DL) models, specifically those employed in tasks such as image recognition and classification, are significantly impacted by the caliber and composition of the datasets utilized for training. Unequal distribution of images in datasets can result in substantial biases, which can affect the performance and fairness of these algorithms. This article examines the consequences of imbalanced datasets on the accuracy of machine learning models and proposes possible solutions to address this issue.

Deep learning models intrinsically depend on data, relying on substantial training data to acquire the fundamental patterns required for making predictions (LeCun et al., 2015). Imbalanced datasets occur when certain classes are overrepresented while others are underrepresented. The model may be biased toward the more frequent classes in such cases. This can decrease performance in underrepresented classes, as the model has fewer learning examples (Buda et al., 2018).

The effect of dataset imbalance is most notable in areas like medical imaging, where certain illnesses may naturally have a lower occurrence rate than others. For example, regarding rare diseases, a limited number of images may be available for training. As a result, models tend to excel in recognizing common diseases but struggle to identify unusual ones (Litjens et al., 2017). This can have immediate implications for clinical decision-making, possibly resulting in incorrect diagnosis.

Dataset imbalance in facial recognition technologies may increase racial biases. Research has indicated that facial recognition systems, which have been primarily trained on datasets mainly consisting of persons of Caucasian descent, exhibit increased inaccuracy rates when attempting to identify individuals from different ethnic origins (Buolamwini and Gebru, 2018). This impacts accuracy and gives rise to ethical considerations regarding the objectivity and comprehensiveness of AI technology.

A prominent method for dealing with imbalanced datasets is data augmentation, which involves artificially boosting the number of data points in underrepresented classes by producing additional data through different transformations (Perez and Wang, 2017). This includes methods such as rotation, scaling, or color modification, which aid in achieving a more balanced dataset without gathering more images.

Another approach involves the production of synthetic data. Generative Adversarial Networks (GANs) can be utilized to generate images that can be incorporated into the training dataset, hence balancing the distribution of classes (Goodfellow et al., 2014). This strategy has proven advantageous when data collecting is difficult, or privacy issues are paramount. Transfer learning is a practical approach for reducing the impact of imbalanced datasets. Transfer learning enables the advantages of deep learning to be utilized even when there is limited data by using a model trained on an extensive and diverse dataset and then fine-tuning it on a smaller and more particular dataset (Pan and Yang, 2010). This approach is particularly advantageous when gathering data is costly or complicated. Ensemble learning techniques, which integrate many models to enhance overall performance, can also efficiently address imbalanced datasets. Ensemble approaches can mitigate the risk of overfitting to the majority class by training many models on different subsets of the data and combining their predictions (Dietterich, 2000).

Although these strategies can help, it is not possible to eliminate dataset imbalance, and it typically needs continuous attention during the model development process. Consistent monitoring and testing in various scenarios guarantee that models perform fairly for all classes. This article examines the influence of imbalanced features on Deep and non-deep models.

## 2 Literature review

Class imbalance emerges when there is significant variation in the number of samples between different classes. This can bias models toward the majority class. Class imbalance occurs when one class has significantly more samples than other classes. This can bias models toward the majority class. The classification of imbalanced data, where there are many more samples of the majority class compared to the minority class, is a challenging problem for deep learning models. Class predictions tend to be biased toward the majority class, resulting in poor accuracy on the minority class. One thesis empirically studies the impact of imbalanced training data distributions on Convolutional Neural Networks (CNNs) performance for image classification (Masko and Hensman, 2015). The CIFAR-10 dataset creates training subsets with different class distributions, ranging from balanced to highly imbalanced. A simple CNN architecture is trained on each subset. Performance is measured by classification accuracy on the held-out CIFAR-10 test set.

Oversampling is also applied to imbalanced subsets to duplicate minority class examples until a balanced distribution is achieved. This allows for evaluating whether oversampling can recover performance lost due to imbalanced training data.

The study reveals that balanced training data yields the best CNN performance, as expected based on prior work. However, performance decreases with increasing imbalance. Highly imbalanced distributions cause the CNN to simply predict the majority class. Under-representations of single classes do not significantly impact overall accuracy, where few classes have only a few cases. The experiments show that oversampling successfully recovers performance lost due to imbalanced training, matching results from balanced training. The study provides empirical evidence that balanced training data and oversampling of imbalanced data are essential for optimizing CNN performance on image classification tasks.

CNNs have achieved great success in computer vision. Still, the following study analyzes how data imbalance affects the performance of Convolutional Neural Networks (CNNs) for image classification tasks. It reveals how imbalanced data's impact is poorly understood (Pulgar et al., 2017). The study focuses on classifying images of traffic signs using CNNs, with datasets containing different levels of class imbalance. Four experiments were conducted with imbalance ratios (IR) of 1/10, 1/5, 1/3, and 1/1 (balanced). A CNN architecture with convolution, pooling, and fully connected layers was trained on 2,030 images and evaluated on 670 images in each experiment. Key metrics like error rate, accuracy, and recall were computed. The results show that the classification performance consistently improves across all metrics as the IR decreases (data becomes more balanced). Error rate drops from 3.3% with IR 1/10 to 1.2% with a balanced dataset. Accuracy and recall also increase in each subsequent experiment.

This confirms the initial hypothesis that imbalance negatively impacts CNN's performance. The authors theorize this may be due to the fully connected layer prioritizing majority classes and convolution filters over-adapting to them during training. Although the study demonstrates that data imbalance is a problem for CNN-based image classification and that balancing training data through techniques like resampling can help address this issue and improve results, further work is needed to understand how imbalance affects CNNs, which the present study tries to address in this paper.

One investigated issue of medical images is that the classification tasks suffer from severe class imbalance, where images of target classes of interest (e.g., certain diseases) only appear in a small portion of the dataset (Zhang, 2019). Consequently, this shares two common issues: Only a small labeled training set is available due to the high cost of manual labeling by experts. Also, the rare and common classes have a high imbalance ratio. The study (Zhang, 2019) refers to these two issues because traditional data augmentation, sampling-based methods, and cost-sensitive learning do not utilize large unlabeled image sets to create large representative training sets. Moreover, the existing active learning methods are not designed for medical image classification tasks with unknown feature representations and class imbalance. To overcome these issues, they (1) propose several real data augmentation methods that utilize unlabeled data to expand small labeled training sets, especially for the rare class. (2) A sensitivity study compares the effectiveness of different data augmentation methods with training sets of varying sizes, varieties, and similarities to the test set. (3) A hierarchical and unified data augmentation method is proposed to efficiently collect a large representative training set for the common class. (4) A novel similarity-based active deep learning framework called SAL is introduced to deal with small labeled training sets and significant class imbalance. The key findings suggest that triplet-based real data augmentation methods outperform other techniques, and SAL achieves near-optimal classification performance with minimal manual labeling effort. The study provides valuable insights on selecting appropriate medical image training sets and offers promising solutions to address class imbalance and small labeled data challenges in medical image classification tasks.

A further study focuses on the issue of insufficient annotated datasets and imbalanced classes when utilizing deep learning to detect lung disease indicators from chest X-rays and CT images (Iqbal et al., 2023). The study introduces a new 3-phase Dynamic Learning (3PDL) technique that adjusts the sampling of the minority class throughout training to achieve class balance. The proposed method is assessed using accuracy, F1 score, precision, sensitivity, and specificity. The model obtains a notable F1 score of 96.83% and a precision of 96.87% on the datasets, showing excellent performance. The study's findings suggest that the 3PDL approach and HFF model are more effective than other methods for dealing with class imbalance and using multi-modality data. The study suggests doing additional tests on more extensive datasets. It highlights the need to address data privacy and bias in using deep learning for medical imaging.

Another approach to tackle the imbalanced data problem is incorporating noise into the feature space of a Convolutional Neural Network (CNN) during the training process (Fan and Lee, 2021). Noise is added to the last extracted feature layer of the CNN to perturb the features and encourage the network to learn more separable representations. A hybrid loss function combining cross-entropy and KL divergence is used. The KL divergence term constrains the noise distribution, preventing it from approaching zero during training. The method is evaluated on DAGM 2007, NEU surface defect, and MNIST (artificially imbalanced). A simple CNN architecture selection method is also presented.

The proposed CNN with added noise (CNNnoise) achieves significantly better accuracy on the minority class than a standard CNN, especially at higher imbalance ratios. CNNnoise maintains over 96% accuracy even at an imbalance ratio of 100. Adding noise allows the network to learn features not existing in the original minority class samples. This helps address the lack of meaningful representations for the minority class due to data imbalance. In a different context, a study aimed to effectively classify imbalanced cloud image data with a class imbalance of more than 20 times using deep learning approaches (Matsuoka, 2021). The authors held a data science competition to design a highly accurate classification model where labeled images of tropical cyclones and other categories were publicly available. The top-performing models in the competition used the following techniques: Deep and wide convolutional neural networks (CNNs) such as ResNet, WideResNet, and PyramidNet to increase representational capability. Data augmentation methods for the minority class include flipping, cropping, and shifting to increase training data. Undersampling of the majority class by including misclassified examples to focus on more complex examples. Ensemble learning by combining models trained with different data sampling techniques. Loss functions accounting for class imbalance, such as focal loss. The top model achieved a precision of 0.6236 when the recall was fixed at 0.8062, improving classification performance by around 65.4% compared to the baseline. Common effective methods included deep CNN architectures, data augmentation, ensemble learning, and test time augmentation. The competition successfully improved the classification of imbalanced cloud image data, showcasing the effectiveness of collaboration between computer science and geoscience fields.

From another perspective, another study aimed to investigate how the performance of imbalance classification models is affected by the joint use of feature selection and data resampling methods (Zhang et al., 2023). Specifically, it compared the performance of two pipelines: feature selection before data resampling (FS+DS) and data resampling before feature selection (DS+FS). The following methodologies were used: 52 publicly available imbalanced datasets from various domains, nine feature selection methods (filters, wrappers, embedded) and six resampling methods (oversampling, undersampling), three classifiers (C4.5, SVM, MLP) to build models on preprocessed data, and finally, the performance was evaluated using metrics like accuracy, G-mean, F1, IBA. Statistical tests like Wilcoxon and Iman-Davenport were used to compare pipelines, and heuristic measures like Rank-Sum were used to recommend top combinations.

The study concluded that both pipelines should be considered for the best model. It provided new insights into their performance with different configurations and recommendations on promising feature selection-resampling combinations. The joint use of these techniques can significantly improve imbalance classification. Lastly, a new method has been developed to improve the accuracy of imbalanced medical datasets using Balanced GAN (BAGAN) to generate synthetic images of minority classes (Asokan, 2021). They develop efficient CNN classifier algorithms for classifying medical images from various datasets. They also compare and evaluate different CNN models on the classification performance of synthetic images generated by BAGAN. The study proposes a methodology with the following steps: (1) traditional data augmentation is applied to enlarge the training dataset, (2) BAGAN is used to generate synthetic images of minority classes to balance the datasets, (3) various CNN models are trained on original and BAGAN-balanced datasets, and (4) classification performance is evaluated and compared between different models.

The study finds that BAGAN can generate high-quality synthetic images that balance the class distribution. CNN classifiers achieve better accuracy when trained on BAGAN-balanced datasets compared to imbalanced original datasets. Finally, an ensemble of CNN classifiers further improves the classification performance.

## 3 Proposed approach

This work examines image classification using traditional features, considering both balanced and unbalanced sets of features. It offers a thorough exploration of various image classification models. The first group of models is designed for datasets with imbalanced class distributions, while the second group works with datasets where classes are balanced. The effectiveness of these models is evaluated by testing them on known datasets and comparing their performance in accurately classifying images. Algorithm 1 shows the Pseudocode for the algorithmic flow of the steps for the proposed approach.

**Algorithm 1:**
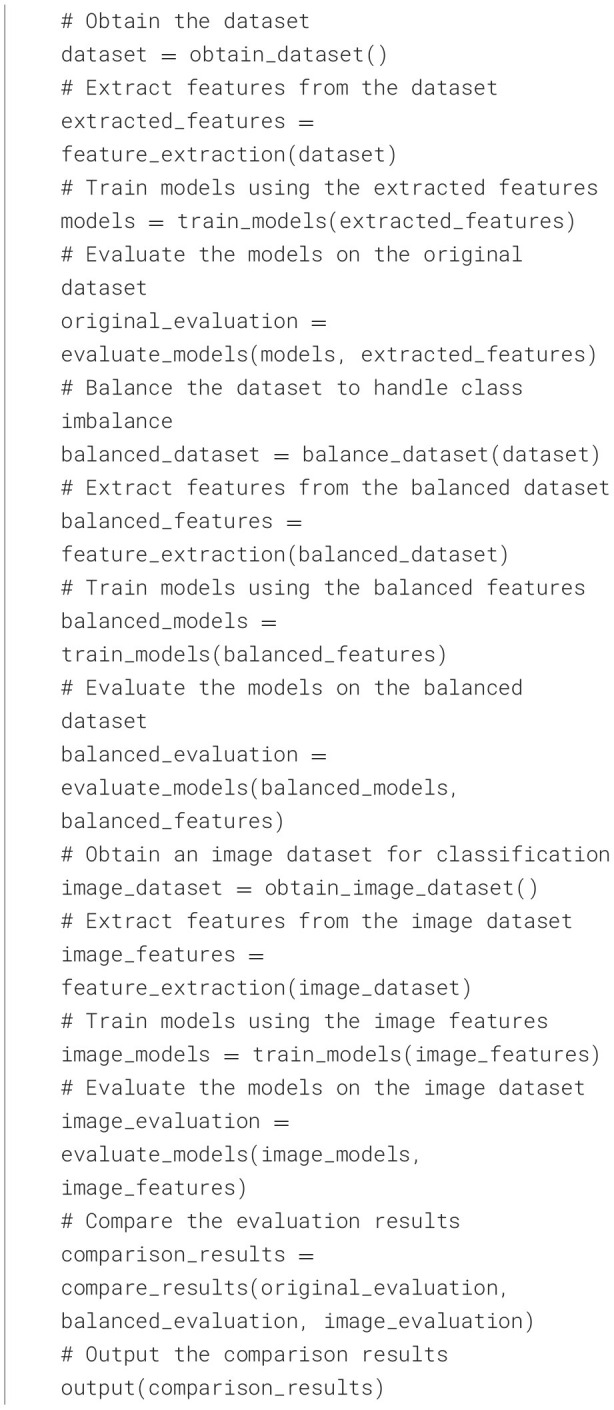
Pseudocode for the algorithmic flow of the steps for the proposed approach.

**Figure 1:**
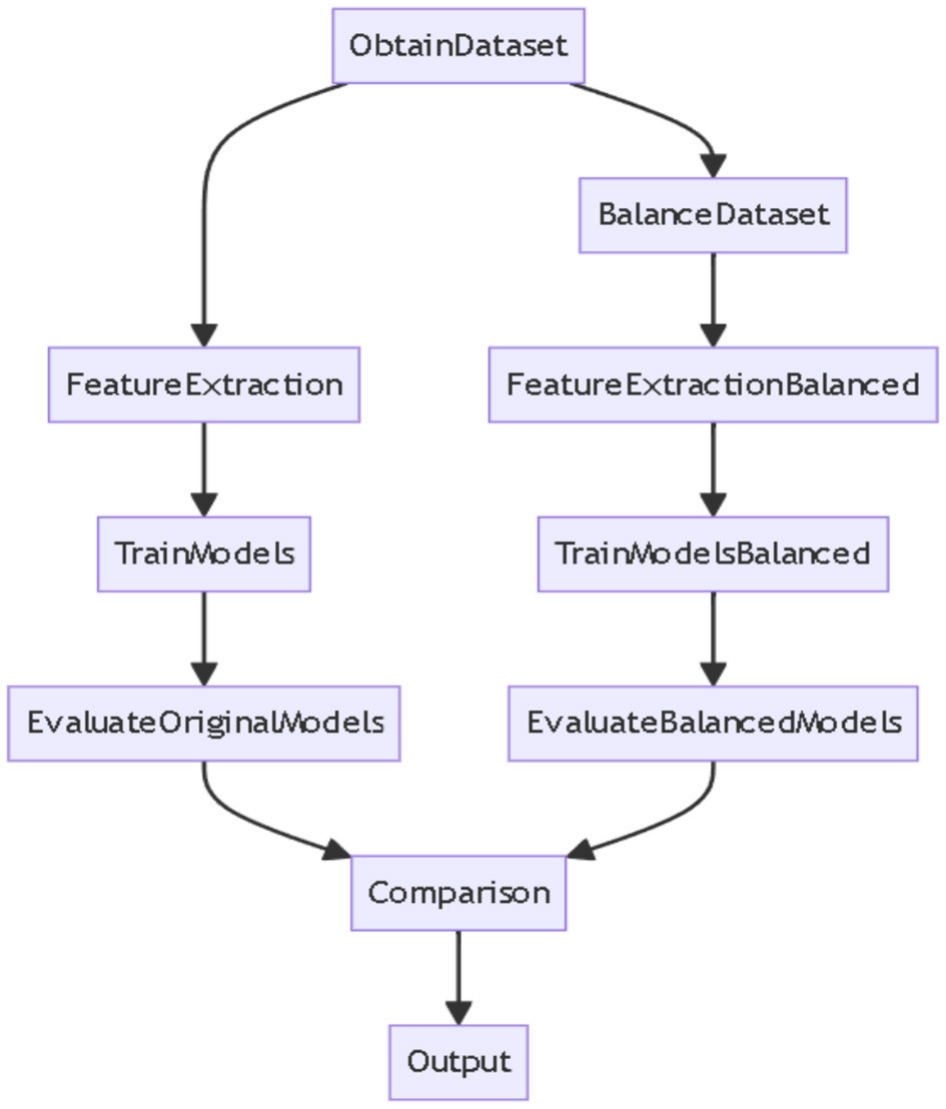
Flow of the steps for the proposed approach.

The following steps are followed to address the imbalanced features in a dataset, see Figure 1. First, a balanced sample is gathered to reduce the imbalance. Then, feature extraction is done using the Auto Color Correlogram method to extract the essential features, making the dataset more accessible for machine learning. After this, ML models are created to classify the data, and their performance is checked to see how well the feature extraction models the data. Also, experiments are executed to handle the imbalanced classes, hoping to improve the overall classification process.

Additionally, multiple models are developed for classifying images. The effectiveness of these models is evaluated to see how well the techniques work. Finally, all the results are compared to determine which strategies are best for dealing with imbalanced features and boosting machine learning performance for image classification.

### 3.1 Model evaluation

Feature classification models (ML models) are selected based on the performance of similar tasks in the state-of-the-art. The Bayesian Network (BN) classifier is a probabilistic graphical model representing variables and their conditional dependencies using a Directed Acyclic Graph (DAG). Multinomial Naive Bayes (NB), a probabilistic classifier designed for discrete data, utilizes Bayes' theorem. Random Forest (RF) is an ensemble learning method for various problems, including classification and regression. It creates multiple decision trees during training, with the outcome determined by either the mode of the classes (for classification) or the average prediction (for regression) of the individual trees. Sequential Minimal Optimization (SMO) is a technique for training Support Vector Machines (SVMs), which are used for classification and regression tasks. Convolutional Neural Networks (CNNs) are deep learning models specialized for analyzing structured grid data and have excelled in numerous computer vision tasks such as image classification, object detection, and segmentation.

### 3.2 Feature balancing

Feature balancing methods, namely the Class Balancer (CB) and the Distribution-Based Balancer (DB), address the issue of imbalanced classes in image datasets.

Feature balancing methods, namely the Class Balancer (CB) and the Distribution-Based Balancer (DB), address the issue of imbalanced classes in image datasets (He and Garcia, 2009). Class imbalance occurs when certain classes are overrepresented while others are underrepresented, which can lead to biased models that perform poorly on minority classes. Balancing techniques aim to mitigate this bias by ensuring the model receives an equitable representation of each class during training.

#### 3.2.1 CB approach

The CB approach adjusts the weights of instances in the dataset so that each class contributes equally to the total weight (Buda et al., 2018). This method works by assigning a weight to each instance inversely proportional to the class frequency, ensuring that no class dominates the training process due to its imbalance (Japkowicz and Stephen, 2002). As a result, the loss function during training is computed in a way that gives equal importance to all classes, regardless of their original proportions in the dataset. The adjustment is made iteratively until the total weight across all classes remains constant, thereby normalizing the influence of each class.

We can represent the above explanation as follows: For the formulation of CB into a more formal mathematical representation, Let D = {x1, x_2_,..., x_*n*_} represent the dataset containing n instances, where each instance x_i_ belongs to a class C_i_ ε C, with class labels C = {C1, C_2_,..., C^*k*^}. The weight of an instance x_i_ is denoted as w_i_. The CB is to reassign weights w_i_ so that each class C_j_ has an equal total weight.

For the initial class distribution computation, Let N_j_ denote the number of instances in class C_j_, such that: N_j_ = | {x_i_ | C_i_ = C_j_} |, for j ε {1, 2,..., k}. The total number of instances in the dataset is n = $\sum_{\text{j}}^{\text{k}}=1$ N_j_.

For the total weight sum preservation, Let W_total be the total weight of the instances in the dataset before balancing: W_total = ∑_i_=1^*n*^ w_i_. The total weight after balancing must remain unchanged: W_total' = W_total.

The goal of assigning equal weight per class is to ensure that each class C_j_ contributes equally to the total weight. Thus, denoting the total weight allocated to each class as W_j_', where:


$$\begin{array}{l} {\text{W}}_{\text{j}}^{\text{'}}=\text{W\_total}/\text{k} \end{array}$$

Each instance x_i_ in class C_j_ must have a weight w_i_ such that: ∑_xi_ ε C_j_ w_i_ = W_j_', for all j ε {1, 2,..., k}.

Thus, the weight assigned to each instance x_i_ ε C_j_ is:


$$\begin{array}{l} {\text{w}}_{\text{i}}\text{'}={\text{W}}_{\text{j}}\text{'}/{\text{N}}_{\text{j}},\text{ for all }{\text{x}}_{\text{i}}\text{ε}{\text{C}}_{\text{j}}. \end{array}$$

Final new weight reassignment: The new weight of an instance x_i_ is then given by:


$$\begin{array}{l} {\text{w}}_{\text{i}}\text{'}=\text{W\_total}/\left({\text{k}}^{*}{\text{N}}_{\text{j}}\right),\text{ for }{\text{C}}_{\text{i}}={\text{C}}_{\text{j}}. \end{array}$$

This ensures that the total weight across the dataset remains constant and each class has an equal total weight contribution.

As such, theoretically, the CB ensures the total sum of weights remains the same while redistributing the weights so that each class contributes equally.

#### 3.2.2 DB approaches (D-GA, D-PO)

On the other hand, the DB approach involves resampling instances using a probabilistic model and, with replacement, each tagged with its respective class label. It operates by following a distribution learned for each combination of attribute and class labels. Specifically, this approach begins by estimating the probability distribution of each class based on its attributes (Liu et al., 2009). Using this distribution, instances are sampled to ensure balanced class representation. This approach is particularly useful in scenarios where certain attribute-class combinations are rare, as it guarantees that the model is exposed to sufficient samples from these combinations during training. Doing so enhances the model's ability to generalize across all classes and reduces the risk of overfitting to the majority classes.

We can represent the above explanation as follows. For the formulation of DB into a more formal mathematical representation, Let C = {C1, C_2_,..., C_k_} be the set of class labels, and A = {A_1_, A_2_,..., A_*m*_} be the set of attributes. Each instance x_i_ is associated with a class label y_i_ ε C and a set of attribute values {a_i_1, a_i2_,..., a_im_} ε A. The Distribution-Based Balancer (DB) approach follows these steps:

For the estimation of probability distribution: For each class C_j_, estimate the conditional probability distribution P(A | C_j_) of attributes A given the class C_j_ from the training data:


$$\begin{array}{l} \text{P}\left(\text{A}|{\text{C}}_{\text{j}}\right)={\text{Π}}_{\text{i}}{=}_{1^{\text{m}}}\text{P}\left({\text{a}}_{\text{i}}|{\text{C}}_{\text{j}}\right) \end{array}$$

where P(a_i_ | C_j_) is the probability of observing attribute a_i_ given class C_j_.

For the sampling of instances: Instances x_i_ are sampled with replacement such that the probability of selecting an instance x_i_ from class C_j_ is proportional to the learned probability distribution P(A | C_j_), ensuring balanced class representation. Balanced Class Representation: The resampling procedure continues until the desired class balance is achieved, meaning the number of instances sampled for each class C_j_ is approximately equal:


$$\begin{array}{l} \text{N}\left({\text{C}}_{\text{j}}\right)\approx \text{N}\left({\text{C}}_{\text{k}}\right)\text{ for all j},\text{ k ε}\left\{1,2,...,\text{k}\right\} \end{array}$$

where N(C_j_) is the number of instances sampled for class C_j_.

Thus, the final dataset used for training or evaluation has a balanced distribution of classes, accounting for the class distribution in the original dataset.

##### 3.2.2.1 D-GA

The Distributed Gaussian (D-GA) method balances a dataset by modeling class attributes using Gaussian distributions and generating synthetic data to achieve class balance. For each class, C_k_ ε C, attributes A follow a Gaussian distribution characterized by the mean μ_k_ and covariance Σ_k_:


$$\begin{array}{l} \text{P}\left(\text{A}|{\text{C}}_{\text{k}}\right)=\text{N}\left(\text{A}|{\text{μ}}_{\text{k}},\;{\text{Σ}}_{\text{k}}\right) \end{array}$$

Whereas, the mean μ_k_ and covariance Σ_k_ are estimated from the data:


$$\begin{array}{l} \;{\text{μ}}_{\text{k}}=\left(1/{\text{N}}_{\text{k}}\right)\sum_{\text{i}}{\text{A}}_{\text{i}}\\ {\text{Σ}}_{\text{k}}=\left(1/\left({\text{N}}_{\text{k}}-1\right)\right)\sum_{\text{i}}\left({\text{A}}_{\text{i}}-{\text{μ}}_{\text{k}}\right){\left({\text{A}}_{\text{i}}-\;{\text{μ}}_{\text{k}}\right)}^{\text{T}} \end{array}$$

New instances are generated by sampling from the Gaussian distribution for each class:


$$\begin{array}{l} {\bar{\text{A}}}_{\text{i}}\sim \text{N}\left({\text{μ}}_{\text{k}},\;{\text{Σ}}_{\text{k}}\right) \end{array}$$

#### 3.2.2 D-PO

The Distributed Poisson (D-PO) method balances a dataset by modeling class attributes using a Poisson distribution and generating synthetic data. For modeling the Class Distributions: For each class C_i_ ε C, the attributes A follow a Poisson distribution with rate λ_i_:


$$\begin{array}{l} \text{P}\left(\text{A}|{\text{C}}_{\text{i}}\right)=\text{Poisson}\left(\text{A}|\;{\text{λ}}_{\text{i}}\right) \end{array}$$

For the estimating the rate λ_i_: It is estimated as the mean of the attribute values:


$$\begin{array}{l} {\text{λ}}_{\text{i}}=\left(1/{\text{N}}_{\text{i}}\right)\sum_{\text{i}}{\text{A}}_{\text{i}} \end{array}$$

The new instances are generated by sampling from the Poisson distribution:


$$\begin{array}{l} {\bar{\text{A}}}_{\text{i}}\sim \text{ Poisson}\left({\text{λ}}_{\text{i}}\right) \end{array}$$

Finally, the dataset is balanced by ensuring each class has the same number of instances:


$$\begin{array}{l} \text{N}\left({\text{C}}_{\text{i}}\right)=\;{{\text{N}}_{\text{ta}}^{\text{r}}}_{\text{γt}} \end{array}$$

Both CB and DB methods are critical in applications involving image classification, where imbalanced datasets are common. These methods can be applied in various domains, such as medical imaging, where minority-class instances (e.g., images of rare diseases) are often underrepresented, leading to potential misdiagnoses (Chawla et al., 2002). By implementing feature balancing techniques, models can be trained to recognize patterns more effectively across all classes, improving overall accuracy and robustness.

## 4 Experimental evaluation

### 4.1 Datasets (DS1, DS2)

The experimental evaluation utilizes the NDPI large video dataset (Avila et al., 2013), represented as DS1. The DS1 is categorized into three groups: acceptable, flagged, and unacceptable. The data obtained via image filtering is used for data sampling. The dataset is effectively structured for three primary reasons: it is divided into three well-defined categories, can be transformed into numerical representations, and encompasses a significant volume of data (up to 40 GB). Consequently, it is well-suited for processing massive amounts of data and doing in-depth analysis using machine learning techniques.

Furthermore, the dataset is especially suitable for experiments that involve imbalanced data classes. In practical situations, datasets commonly show class imbalances, wherein specific categories (such as “acceptable”) have a notably higher number of instances compared to others (such as “flagged” or “unacceptable”). The dataset's classification into acceptable, flagged, and unacceptable categories enables the investigation of approaches to address unbalanced data, such as resampling techniques, cost-sensitive learning, and sophisticated algorithms designed to operate effectively in class imbalances. Due to these characteristics, it is suitable for studying and developing machine-learning solid models that can efficiently handle and learn from imbalanced data distributions.

The Retinopathy image dataset (represented as DS2) retrieved from Kaggle plays a key role in detecting diabetic retinopathy (DR), a significant cause of blindness. It consists of retina images taken through fundus photography and is used to train machine learning models to identify and classify the severity of DR. The images are labeled with severity levels, ranging from No DR (0) to Proliferative DR (4), with other stages like Mild DR (1), Moderate DR (2), and Severe DR (3). The dataset includes over 35,000 images, with around 35,000 in the training set and about 5,000 in the test set. These images help train models and spot subtle changes in the retina that may not be easy for the human eye to detect. Preprocessing steps like resizing, normalization, and augmentation (like rotating and flipping images) are applied to improve model accuracy. The dataset is challenging, especially in detecting early-stage DR. It's widely used for automated diagnosis and testing of machine learning models in tasks like image classification, segmentation, and object detection.

### 4.2 Experimental setup

The experimental evaluation aims to build several models for classifying images into their categories and then evaluate the classification performance. The Auto Color Correlogram is used to extract the features. BN, Multinomial Naive Bayes, Simple Logistic, Random Forest, and SMO are used for model creation.

The First set of experiments involves generating the model with an unbalanced original feature set. The second set of experiments balances classes in the image dataset and then applies the proposed models initially applied to the original dataset. The Third set of experiments uses the CNN model to classify images according to their category and then evaluate the classification performance. In addition, in this experiment, an auto-balancing function is used.

The CNN model consists of four Convolution and Pooling layers, each paired with a ReLU activation function. After these layers, there's a flattened layer, which bridges the Convolution and Dense layers. The Dense layers serve as the output layers, employing the Softmax function to assign probabilities to each class. Three Dropout layers are integrated into the model to mitigate overfitting, preventing excessive reliance on specific features when connected to the flattened layer.

## 5 Evaluation and analysis

In this section, we present the experimental evaluation and report the performance.

### 5.1 Evaluation of balanced and unbalanced classical features

Table 1 shows the balancing techniques used for evaluation. Four balancing techniques were examined and compared with the original dataset.

**Table 1:** Balancing techniques.

**Approach**	**Definition**
IB	Imbalance: performance with the original imbalanced dataset
MB	Manual balance: performance after manually balancing the dataset
CB	Class balancer: utilizing automated class balancing techniques
D-GA	Distributed Gaussian: performance using a Gaussian distribution approach to balance the dataset
D-PO	Distributed Poisson: performance using a Poisson distribution approach to balance the dataset

The original imbalance dataset is a base for the other four techniques used in different classification algorithms. In manual balancing, the dataset is manually balanced by excluding features that make the dataset unbalanced. A class balancer adjusts the weights of instances in the dataset so that each class contributes equally to the total weight. It ensures that no class dominates the training process due to its imbalance. Lastly, the Distribution-Based Balancer involves resampling instances with replacement, each tagged with its respective class label. Instances are sampled based on the learned distribution to ensure a balanced representation of classes during training and the corresponding modeling algorithm.

#### 5.1.1 DS1 evaluation

**Figure 2:**
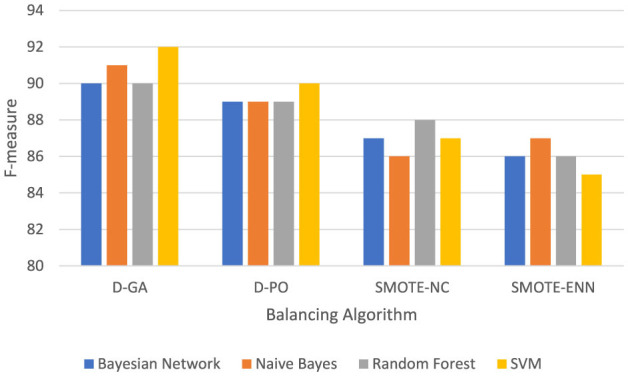
F-measure of DS1 evaluation. Visualizing only D-GA, D-PO, and SMOTE for better visual comparison.

Table 2 and Graph of DS1 in Figure 2 present the F-measure values for various models applied to the DS1 dataset, balanced using different approaches. The F-measure balances precision and recall, with higher values indicating better model performance. The Bayesian Network performs best with the D-GA method, achieving a strong F-measure of 90, followed by D-PO at 89, SMOTE-NC at 87, and SMOTE-ENN at 86. This suggests that D-GA is the most effective balancing technique for this model. Naive Bayes also performs best with D-GA (91), slightly outperforming the Bayesian Network, but sees a drop in performance with D-PO (89) and a more significant decline with SMOTE-NC (86) and SMOTE-ENN (87). Random Forest follows a similar trend, with its highest F-measure (90) achieved using D-GA, but its performance drops slightly with D-PO (89) and SMOTE-NC (88) and more noticeably with SMOTE-ENN (86). SVM stands out as the top performer overall, with a peak F-measure of 92 using D-GA, followed by 90 with D-PO, and a noticeable decrease with SMOTE-NC (87) and SMOTE-ENN (85). Across all models, D-GA consistently yields the best results, indicating its effectiveness in balancing the dataset. While D-PO also performs well, SMOTE-NC and SMOTE-ENN result in lower performance, with SMOTE-NC slightly outperforming SMOTE-ENN. In conclusion, D-GA is the best method for balancing the dataset, with SVM delivering the top performance overall, followed by Naive Bayes, Bayesian Network, and Random Forest.

**Table 2:** F-measure for DS1 evaluation.

**Model**	**IB**	**MB**	**CB**	**D-GA**	**D-PO**	**SMOTE-NC**	**SMOTE-ENN**
Bayesian network	80	76	81	90	89	87	86
Naive Bayes	70	67	70	91	89	86	87
Random forest	84	82	86	90	89	88	86
SVM	88	87	89	92	90	87	85

#### 5.1.2 DS2 evaluation

**Figure 3:**
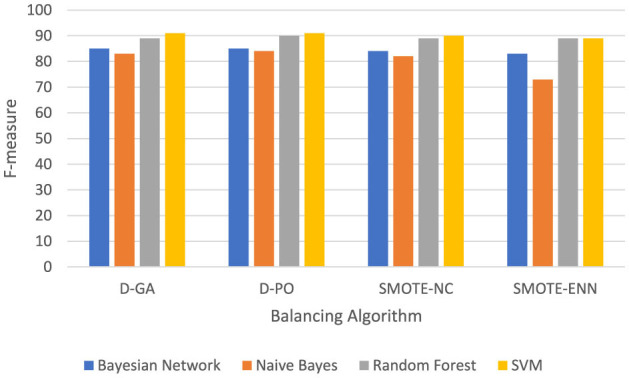
F-measure of DS2 evaluation. Visualizing only D-GA, D-PO, and SMOTE for better visual comparison.

Table 3 and the DS2 graph in Figure 3 show the F-measure values for different models applied to the DS2 dataset, with data balanced using various methods. The Bayesian Network performs consistently across all methods, achieving its highest F-measure (85) with both D-GA and D-PO and slightly lower values with SMOTE-NC (84) and SMOTE-ENN (83), showing minimal sensitivity to the balancing technique. Naive Bayes performs best with D-PO (84), just edging out D-GA (83), but it drops off more significantly with SMOTE-NC (82) and especially with SMOTE-ENN (73), suggesting that this model struggles with SMOTE-ENN. Random Forest is the most consistent, showing an F-measure of 89 for D-GA, SMOTE-NC, and SMOTE-ENN and a slight improvement with D-PO (90), meaning it performs well regardless of the balancing method. SVM performs the best overall, reaching an F-measure of 91 with both D-GA and D-PO, with a slight drop to 90 with SMOTE-NC and 89 with SMOTE-ENN, indicating that it benefits most from D-GA and D-PO. In conclusion, D-GA and D-PO are the most effective balancing methods across most models, with SVM standing out as the best-performing model overall. Random Forest is robust to different balancing techniques, while Naive Bayes performs the worst with SMOTE-ENN.

**Table 3:** F-measure for DS2 evaluation.

**Model**	**IB**	**MB**	**CB**	**D-GA**	**D-PO**	**SMOTE-NC**	**SMOTE-ENN**
Bayesian network	83	80	83	85	85	84	83
Naive Bayes	72	70	72	83	84	82	73
Random forest	86	81	87	89	90	89	89
SVM	89	83	88	91	91	90	89

### 5.2 Evaluation of the deep features

The findings of deep learning investigations implementing Convolutional Neural Networks (CNN) on IB and CB datasets show significant observations and provide clarity on the influence of data balancing on model performance. The performance is evaluated using the F-measure metric, as indicated in Table 4.

**Table 4:** Summary of CNN model results.

**Model (Epoch)**	**IB**	**CB**
CNN20	86	84
CNN40	88	90
CNN60	90	88
CNN100	91	93
CNN150	92	94
CNN200	92	96

We start the overall evaluation with increasing epoch sizes. Initially, with the 20 epochs, the model trained on an imbalanced dataset performed slightly better, achieving an F-measure of 86, compared to 84 for the model trained on balanced data. This slight increase in performance for the imbalanced data may be due to the model being more diverted to the existing correlated feature distribution of the classes. However, from 40 epochs onward, the balanced data model begins to outperform its imbalanced counterpart, with an F-measure of 90 compared to 88 at 40 epochs. This trend continues till the 100 epochs, where the balanced data model achieves an F-measure of 93, compared to 91 for the imbalanced data model, indicating that the benefits of class balancing become more evident with additional training.

Interestingly, at 60 epochs, there was a temporary shift where the imbalanced data model achieved a higher F-measure of 90 compared to 88 for the balanced data model. This anomaly could be due to the model on imbalanced data finding a local optimum that momentarily boosts its performance. Despite this, the overall trend favors the balanced data model, which consistently shows superior performance in the later stages of training. With the 150 epochs, the balanced data model achieved an F-measure of 94, while the imbalanced data model lagged at 92. This gap widened at 200 epochs, where the balanced data model reached the highest F-measure of 96, significantly outperforming the imbalanced data model, which remained at 92.

The CNN evaluation results highlight the importance of balancing classes when training deep learning models. Models trained on imbalanced data may achieve good performance initially. On the other hand, models trained on balanced data keep getting better. This shows that balancing classes makes models more accurate and reliable. Balancing classes contributes to increasing performance, especially when dealing with datasets with large imbalances among classes.

### 5.3 Results analysis

The findings indicate a significant performance improvement, ranging from 10 to 20% when employing distribution-based balancers before image classification. Dataset balancing significantly improves the performance of all models compared to using an imbalanced dataset, highlighting the crucial role of dataset balancing in achieving high model accuracy. Out of the various methods, Distributed Gaussian (D-GA) and Distributed Poisson (D-PO) consistently yield the highest performance across all models, with a particular advantage for Random Forest (RF) and SVM models. The Naive Bayes (NB) model exhibits the most negligible improvement when manual balancing is applied, indicating that it is less affected by variations in class distribution or that manual techniques are less impactful for this particular model.

The experiments demonstrate that utilizing practical balancing approaches, such as Distributed Gaussian and Distributed Poisson, to tackle dataset imbalance can significantly enhance model performance. This improvement is particularly noticeable in models such as Random Forest and SVM, which demonstrate substantial benefits using these techniques. This investigation emphasizes the crucial significance of dataset preparation in developing efficient machine-learning models, especially when dealing with imbalances in class distribution.

Experiments involving deep learning and convolutional neural networks (CNNs) have demonstrated that models' performance is greatly influenced by the balancing of data in imbalanced (IB) and class-balanced (CB) datasets. The analysis presented highlights the significance of class balancing while training deep learning models. It demonstrates that models trained on balanced data regularly achieve superior performance during the later stages of training.

### 5.4 Qualitative analysis

In the experimental evaluation of the previous section, we see that the D-GA and D-PO increase the accuracy of the classifiers used without balancing. With this increase, we constitute a Qualitative question*: Can the D-GA and D-DPO increase the classification accuracy for other N classifiers*? To answer this qualitative question, we select the Neural Network, Gradient Boosting, AdaBoost, and Logistic Regression from the set of classifiers. These classifiers are chosen based on their good overall performance in the state-of-the-art. Table 5 shows the Comparative classifiers, where IB is accuracy without balancing, D-GA is calculated after balancing with Distributed Gaussian, and D-PO is calculated after balancing with the Distributed Poisson approach.

**Table 5:** Comparative classifiers; the experimental evaluation for the qualitative solution.

**Model**	**IB**	**D-GA**	**D-PO**
Neural network	82	88	89
Gradient boosting	80	87	87
AdaBoost	68	73	72
Logistic regression	72	80	80.5

**Figure 4:**
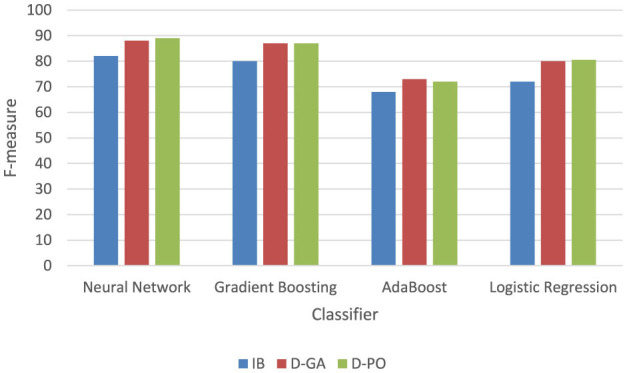
Comparative classifiers; the experimental evaluation for the qualitative solution.

The experimental evaluation of different classifiers for a qualitative solution, as presented in Figure 4, reveals a clear hierarchy in performance across three metrics: IB, D-GA, and D-PO. The Neural Network classifier emerges as the top performer, consistently achieving the highest scores across all metrics (IB: 82, D-GA: 88, D-PO: 89). Following closely is the Gradient Boosting classifier, which demonstrates strong performance with only slightly lower scores (IB: 80, D-GA: 87, D-PO: 87). These two classifiers significantly outperform the others, establishing themselves as the most effective options for the given qualitative solution.

In contrast, AdaBoost and Logistic Regression classifiers show notably lower performance. AdaBoost ranks third with scores of 68, 73, and 72 for IB, D-GA, and D-PO, respectively, while Logistic Regression trails behind with scores of 72, 80, and 80.5. The consistent ranking across all three metrics underscores the reliability of this performance assessment. This clear differentiation in performance suggests that for this particular qualitative solution, the Neural Network and gradient-boosting classifiers are the most suitable choices, with the Neural Network having a slight edge. However, the choice between these top two may also depend on factors such as computational resources and specific application requirements.

Table 4 thus shows that D-GA and D-PO help increase the accuracy of *N* classifiers. This, therefore, validates the qualitative question that sampling with optimal algorithms can improve the classification performance in almost all classifiers, showing the efficacy of the D-GA and the D-PO.

### 5.5 Statistical significance

For statistical significance evaluation and better visualization and understanding of the D-GA and D-PO vs. SMOTE, we present the results in the graph for both the DS1 and DS2 datasets. We omit other approaches for this visualization because their performance is already lower than the D-GA, D-PO, and SMOTE alternatives.

#### 5.5.1 Dataset DS1

**Figure 5:**
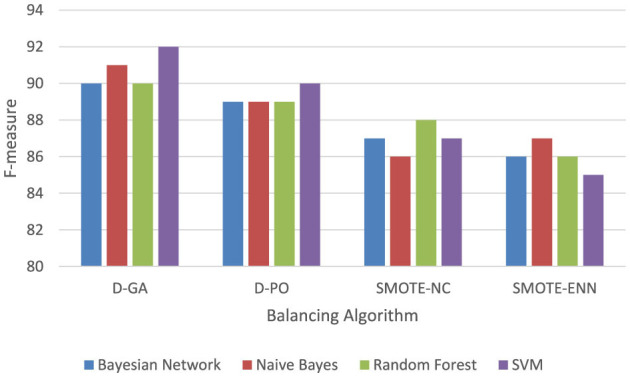
F-measure of DS1 evaluation. Visualizing only D-GA, D-PO, and SMOTE for statistical significance.

The Graph in Figure 5 shows that the D-GA is the best method for balancing the dataset DS1 compared to the SMOTE alternatives.

As can be seen from the results in the graph, the D-GA outperforms the D-PO, SMOTE-NC, and SMOTE-ENN. Therefore, for statistical evaluation, we evaluate the D-GA with the SMOTE-NC and SMOTE-ENN for the DS1. We select D-GA only because D-GA already outperforms other approaches, and thus, we are only interested in evaluating it with the SMOTE alternatives.

##### 5.5.1.1 D-GA vs. SMOTE-NC

**Figure 6:**
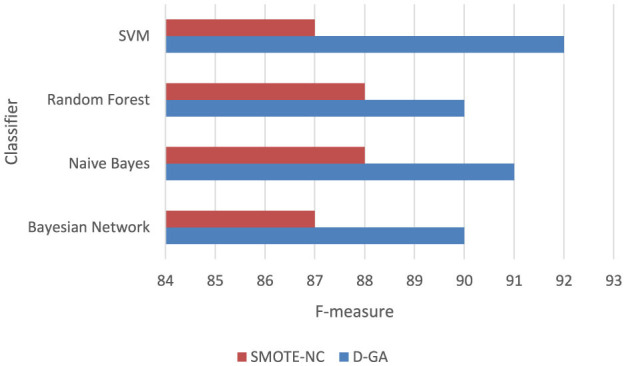
F-measure DG-A vs SMOTE_NC.

To confirm whether D-GA truly outperforms SMOTE-NC, we performed a paired *t*-test on the F-measure values from four models (Table 6, Figure 6). This test is helpful because it directly compares each model's performance under both balancing techniques, helping us determine if the difference is real or just due to chance. The results showed a t-statistic of 5.166 and a *p*-value of 0.014. Since the *p*-value is well below the standard cutoff of 0.05, we can confidently say that the difference in performance is statistically significant. D-GA consistently had a higher F-measure than SMOTE-NC in every tested model, meaning its advantage is significant on DS1. These results suggest that D-GA is the better balancing method, offering a clear and reliable improvement in classification performance. The graph below also confirms this.

**Table 6:** F-measure of the D-GA and SMOTE-NC is shown here to calculate the statistical significance.

**Model**	**D-GA**	**SMOTE-NC**
Bayesian network	90	87
Naive Bayes	91	88
Random forest	90	88
SVM	92	87

##### 5.5.1.2 D-GA vs. SMOTE-ENN

For this set, we select D-GA only because D-GA already outperforms other approaches, and thus, we are only interested in evaluating it with the SMOTE alternatives.

**Figure 7:**
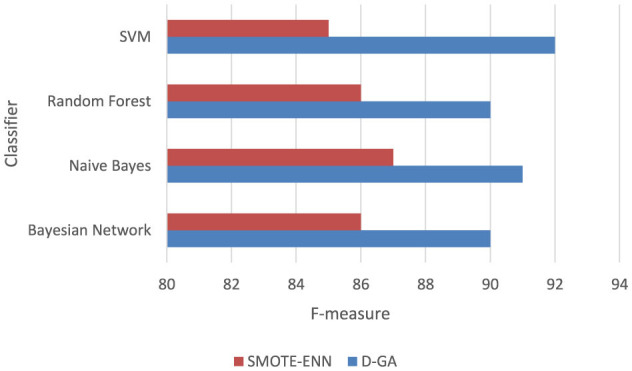
F-measure DG-A vs SMOTE-ENN.

For D-GA vs. the SMOTE-ENN (Table 7, Figure 7), a paired *t*-test is conducted to compare the F-measure performance of D-GA and SMOTE-ENN across four models. The mean difference between the two approaches was 4.75, with a standard deviation of 1.5, leading to a *t*-value of 6.33 for 3 degrees of freedom. The critical *t*-values at α = 0.05 and α = 0.01 are 2.353 and 4.541, respectively, meaning the computed *t*-value far exceeded both thresholds. This resulted in a *p*-value well below 0.01, indicating a highly significant difference. As a result, we reject the null hypothesis and conclude that D-GA significantly outperforms SMOTE-ENN. The consistent improvement across all models, particularly in SVM (+7 points), indicates that D-GA is the superior approach for balancing on DS1.

**Table 7:** F-measure of the D-GA and SMOTE-ENN is shown here to calculate the statistical significance.

**Model**	**D-GA**	**SMOTE-ENN**
Bayesian network	90	86
Naive Bayes	91	87
Random forest	90	86
SVM	92	85

#### 5.5.2 For dataset DS2

**Figure 8:**
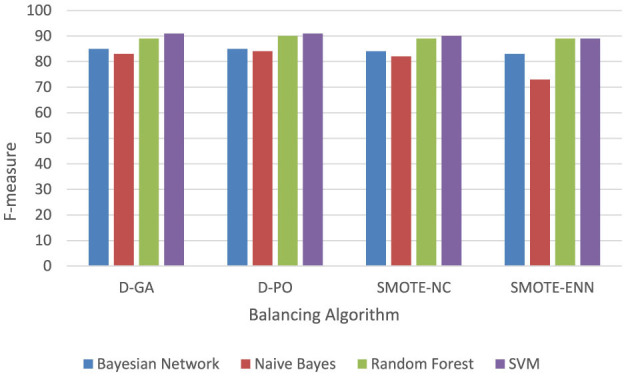
F-measure of DS2 evaluation. Visualizing only D-GA, D-PO, and SMOTE for statistical significance.

The following graph in Figure 8 shows that the D-GA is the best method for balancing the dataset DS2 compared to the SMOTE alternatives.

##### 5.5.2.1 D-GA vs. SMOTE-NC

**Figure 9:**
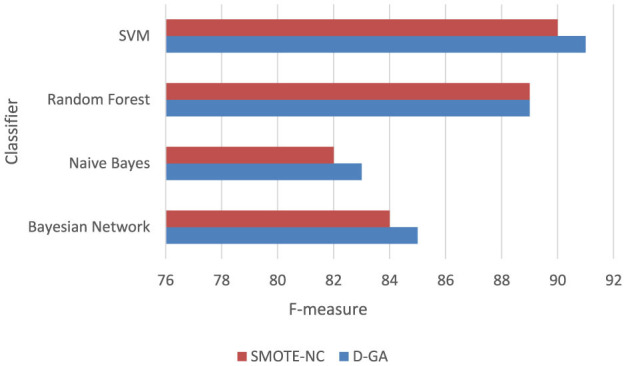
F-measure DG-A vs SMOTE-NC.

We conducted a paired *t*-test to see if D-GA performs significantly better than SMOTE-NC regarding F-measure (Table 8, Figure 9). Looking at the differences between the two methods across four models, we found an average difference of 0.75 with some variation (a standard deviation of 0.661). The statistical test resulted in a *t*-value of 2.27 and a *p*-value of 0.06. While D-GA showed a slight advantage over SMOTE-NC, the *p*-value is just above the typical significance threshold of 0.05. This thus signifies a slight performance improvement over the SMOTE-NC.

**Table 8:** F-measure of the D-GA and SMOTE-NC is shown here to calculate the statistical significance.

**Model**	**D-GA**	**SMOTE-NC**
Bayesian network	85	84
Naive Bayes	83	82
Random forest	89	89
SVM	91	90

##### 5.5.2.2 D-GA vs. SMOTE-ENN

**Figure 10:**
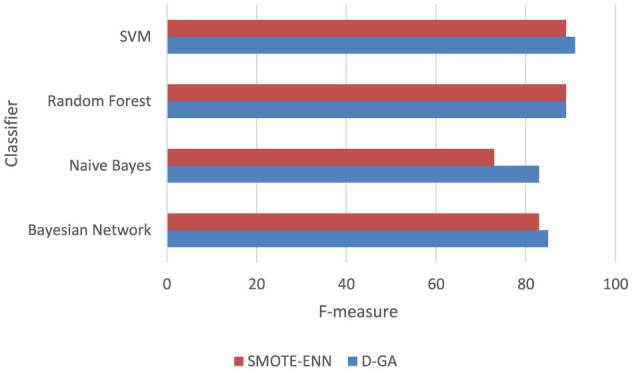
F-measure DG-A vs SMOTE-ENN.

A paired *t*-test was conducted to compare the F-measure performance of D-GA and SMOTE-ENN across four models, using a relaxed significance level of α = 0.10 (Table 9, Figure 10). The mean difference was 3.5, with a standard deviation of 4.43, resulting in a *t*-value of 1.58 for 3 degrees of freedom. The critical *t*-value at α = 0.10 is 1.638, meaning the computed *t*-value was slightly below the threshold. This yielded a *p*-value of ~0.11, indicating borderline statistical significance. While the difference is insignificant at α = 0.10, the results suggest a clear trend favoring D-GA, particularly in models like Naïve Bayes, where it outperformed SMOTE-ENN by 10 points.

**Table 9:** F-measure of the D-GA and SMOTE-NC is shown here to calculate the statistical significance.

**Model**	**D-GA**	**SMOTE-ENN**
Bayesian network	85	83
Naive Bayes	83	73
Random forest	89	89
SVM	91	89

### 5.6 Quantitative analysis

To provide more insights into the challenges of imbalanced data analysis, different approaches are introduced in this section as follows:

I. **Dimensionality Analysis:** Investigate the impact of dimensionality reduction (e.g., using PCA).

We reduce the dimensionality of the data using **Principal Component Analysis (PCA)** and observe how it affects the performance of the four classifiers (Bayesian Network, Naive Bayes, Random Forest, and SVM).

The observations on the classifiers' performance show that Random Forest and SVM models maintain relatively high accuracy as dimensionality increases. In contrast, Naive Bayes shows a drop in performance as the number of components increases.

II. **Noise Intensity Analysis:** Introduce controlled noise to see how the classifiers behave.

To analyze the impact of **noise intensity**, we introduced varying levels of random noise into the dataset and observed how the classifiers behaved.

The experiments performed varying noise levels (0.1, 0.5, and 1.0) on the dataset. With the noise introduction, the classifiers show some drop in performance as noise intensity increases, though Random Forest and SVM remain relatively robust. Naive Bayes shows consistent performance across noise levels but at a lower accuracy overall.

III. **Outlier Impact Analysis:** Introduce outliers and measure their effect on classifier performance.

To analyze the impact of **outliers**, we introduce extreme values into the dataset and observe how each classifier's performance is affected. Naive Bayes depicts a significant drop in accuracy, suggesting that it is sensitive to the presence of outliers. In contrast, Random Forest and SVM remain relatively robust, with only slightly reduced accuracy.

IV. **Non-uniform Distribution Impact:** Adjust the data distribution and evaluate classifier robustness.

To analyze the impact of a **non-uniform data distribution**, we modified the dataset so that some regions of the feature space are more densely populated than others, creating a skewed or imbalanced distribution.

Naive Bayes experienced a notable drop in performance, indicating sensitivity to the non-uniform distribution. Random Forest and SVM remained relatively robust, with their accuracy only slightly impacted.

## 6 Conclusions

The exponential growth of image and video data motivates practical real-time content-based searching algorithms. This article thus analyzed the classification paradigm based on image features with balanced and unbalanced scenarios. The results showed that models perform better when using balanced datasets than imbalanced ones, highlighting the importance of dataset balancing for model accuracy. Among the techniques evaluated, D-GA and D-PO are the most effective, particularly in enhancing the performance of the RF and the SVM models. The NB showed the most minor improvement from manual balancing, depicting that it is either less influenced by class distribution or that manual balancing methods are less effective. Future research should continue exploring innovative balancing methods and their applications across various classification algorithms to enhance the accuracy and reliability of image-based classifiers in real-time content searching.

## Data Availability

The original contributions presented in the study are included in the article/supplementary material, further inquiries can be directed to the corresponding author.
